# Impact of bio-processing treatments on the nutritional and anti-diabetic enzyme inhibitory properties of black wheat, barnyard millet, and black soybean

**DOI:** 10.3389/fnut.2025.1554993

**Published:** 2025-03-18

**Authors:** Krati Goel, Archana Kushwaha, Anuradha Dutta, Satish Kumar Sharma, Navin Chandra Shahi, Dinesh Chandra Joshi, Anuradha Bhartiya, Pranshi Gupta

**Affiliations:** ^1^Department of Food and Nutrition, College of Community Science, G.B. Pant University of Agriculture and Technology, Pantnagar, Uttarakhand, India; ^2^Department of Food Science and Technology, College of Agriculture, G.B. Pant University of Agriculture and Technology, Pantnagar, Uttarakhand, India; ^3^Department of Processing and Food Engineering, MCAET, Acharya Narendra Dev University of Agriculture and Technology, Ayodhya, Uttar Pradesh, India; ^4^Division of Crop Improvement, ICAR-VPKAS, Almora, Uttarakhand, India

**Keywords:** diabetes, soaking, germination, glycemic index, enzyme inhibitory activity, protein digestibility

## Abstract

**Objective:**

The present study assessed the impact of different bio-processing treatments on the *in vitro* protein digestibility (IVPD) and inhibitory properties against *α*-amylase and α-glucosidase of whole grains *viz.* black wheat (BW), barnyard millet (BM), and black soybean (BS) using at initial optimized conditions including soaking and germination.

**Method:**

Nutritional analysis of grains was done using standard AOAC methods and *in vitro* models were used for assessing the anti-diabetic properties.

**Results:**

Nutritional analysis of the grains revealed significant differences in various parameters. BS having higher levels of moisture, ash, crude protein, crude fat, and physiological energy value compared to BM and BW. Optimization of soaking (0–24 h) and germination (0–72 h) times showed significant improvements in *in-vitro* protein digestibility (IVPD), trypsin inhibitory activity (TIU/mg), and glycemic index (GI) across all samples. Considering the management of hyperglycemia, BW and BM do not require any prior processing to be utilized for the development of food products. However, for BS, soaking followed by germination for 48 h proved to be an effective processing, that resulted in an IVPD of 68.706% and a GI of 51.03, with a TIU/mg of 23.166. Soaking reduced *α*-amylase and α-glucosidase inhibition, while germination increased inhibitory activity significantly (*p* < 0.05).

**Conclusion:**

These findings highlighted the potential benefits of incorporating BW, BM, and BS into the diet for enhanced nutrient intake and better management of hyperglycemia considering the GI and inhibitory activity of *α*-amylase, α-glucosidase, and trypsin enzymes.

## 1 Introduction

In the realm of nutrition, whole grains (WG) stand out as vital components of a healthy diet. Comprising the outer bran, germ, and inner endosperm, these grains are abundant in dietary fiber, antioxidants, and essential micronutrients (1). Various researches including human trials and prospective cohort studies increasingly highlights the role of whole grains in reducing fat mass, increasing resting metabolic rate, promoting negative energy balance, increasing insulin sensitivity, managing hyperglycemia, improving the lipid profile, and reducing systemic inflammation (1, 2).

Diet plays a crucial role in managing hyperglycemia. Key dietary factors, including the content of carbohydrates, fiber, and protein, can significantly influence the glycemic index (GI) and insulin secretion (3). The GI is a measure that ranks foods based on how quickly glucose is released into the bloodstream after consumption (3, 4). Foods are categorized into high-GI (> 70), intermediate-GI (55–70), and low-GI (< 55) based on their effects on blood glucose levels (5). Incorporating low-GI foods into daily diets can be particularly beneficial especially for the management of blood glucose levels (6). For instance, pigmented grains like black wheat (BW), millets such as barnyard millet (BM), and legumes like black soybean (BS) can help regulate postprandial insulin and glucose levels. These foods fall under low GI (<55) category among other grains that are part of their respective classes of cereals, millets and legumes. This cause only moderate or low increases in glucose after intake, followed by a gradual return to baseline levels, thereby aiding in maintaining stable glucose levels (7). Supporting this, a recent network meta-analysis of 56 trials demonstrated that low-GI diets significantly reduced both glycated hemoglobin (HbA1c) and fasting blood glucose (FBG) compared to control diets (8).

Another approach to controlling postprandial glycemia involves slowing down carbohydrate digestion. This can be achieved by inhibiting the activity of enzymes like *α*-amylase and α-glucosidase, which are responsible for breaking down starches into glucose (9, 10). *α*-Amylases, found in the salivary glands and pancreas, break down starches into disaccharides and oligosaccharides. These are further converted into glucose by α-glucosidases located in the small intestine. By inhibiting these enzymes, the rate of starch digestion slows, which can help manage postprandial hyperglycemia.

However, managing diabetes is not solely about dietary components. The structural properties of raw materials, the types of processing methods used, and the structure of the final food products can all impact the GI and insulin secretion (3). Common processing techniques such as soaking and germination induce a range of qualitative changes in raw foods, affecting their physical properties, nutritional composition, and starch characteristics. Despite their significance, there is limited data on how these processing methods affect low-GI foods. Also, while studies have explored the benefits of bio-processing treatments like germination and soaking on individual cereals and legumes, limited research exists on their combined impact on nutrient digestibility and enzyme inhibitory activity in diverse genotypes.

Therefore, to address this gap, the present study focuses on optimizing the processing parameters and examining how soaking and germination impact the nutritional and anti-diabetic properties of low-GI foods like BW, BM, and BS. Also, combined impact on nutrient digestibility and enzyme inhibitory activity in diverse genotypes such as BW, BM and BS was studied in the present research. This bridges that gap by providing genotype-specific insights into bio-processing benefits. Through this research, we aim to enhance our understanding of how processing methods can be leveraged to improve the management of diabetes and contribute to better health outcomes.

## 2 Materials and methods

### 2.1 Raw materials

BW line [NABIMG-11-Black (BW/2* PBW621; IC0620916; INGR17003), a wheat (*Triticum aestivum*) germplasm with black grain color] was procured from the local market of Uchaiti village, Moradabad, Uttar Pradesh, India. BM (VL172) and BS (VL Bhat 201) were procured from Vivekananda Parvatiya Krishi Anusandhan Sansthan (VPKAS), Almora, Uttarakhand, India. The glucose assay kit was purchased from Megazyme International Ireland Ltd. (Wicklow, Ireland). All other analytical grade reagents were purchased from Sisco Research Laboratories Pvt. Ltd. (SRL), Mumbai, India.

### 2.2 Cleaning and processing

**Figure 1:**
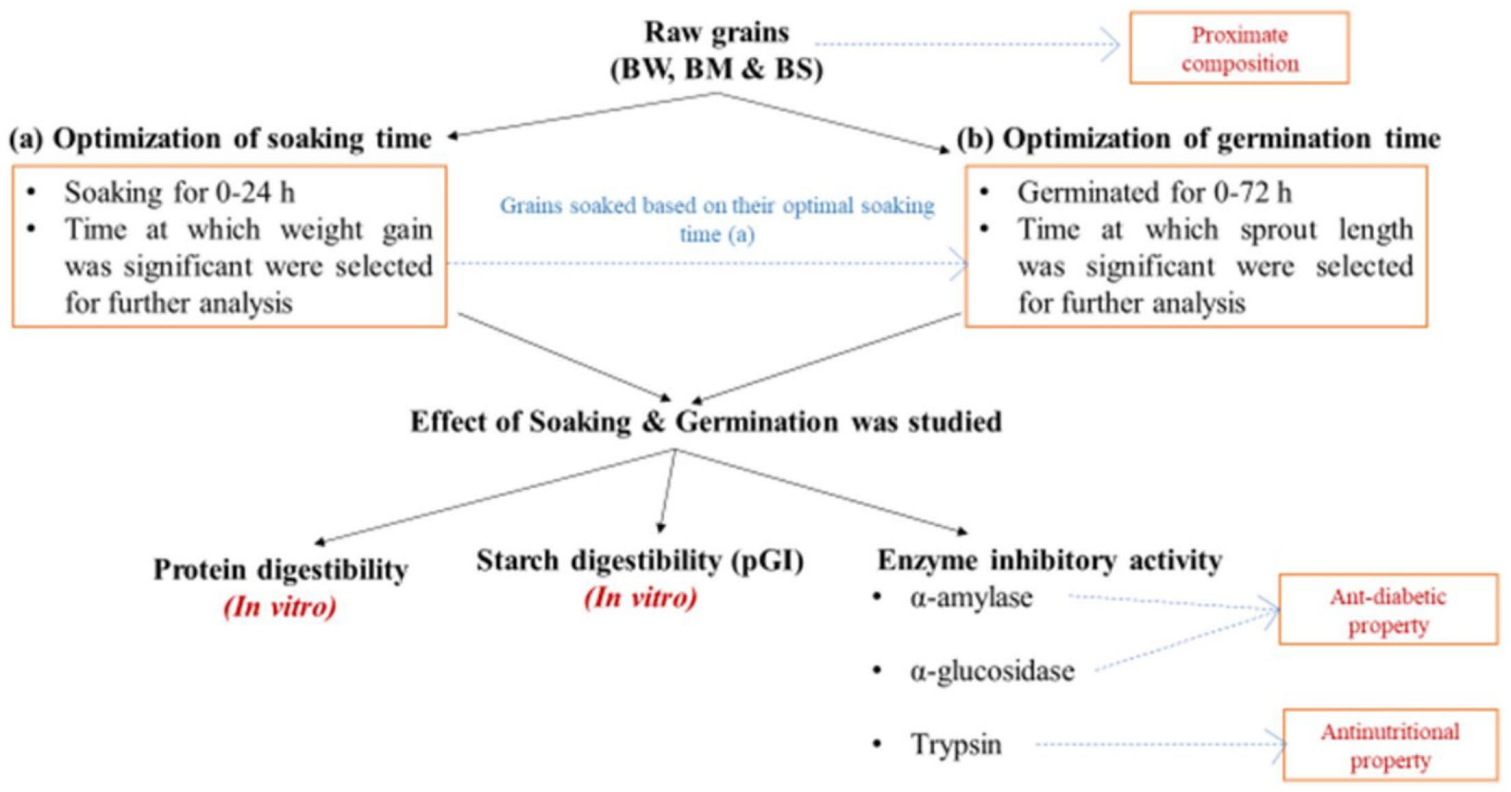
Schematic representation to study the effect of processing on BW, BM and BS.

All extraneous materials, including broken seeds, dust, stones, and fragments of metal and glass, were removed manually from the grains. BW, BM and BS were adjusted to a moisture content of 10 ± 1%. Samples were soaked at 30 ± 5°C (RH > 80%) and weighed after a gap of every 1 h until consistent weight gain was observed. Germination was carried out at 30 ± 5°C (RH > 80%) until a non-significant increase in sprout length was observed. Soaked and germinated samples were dried at 40 ± 5°C for 24 h. The samples were ground to make flour using a mixer grinder (INALSA, MG Compact Lx, B00ID6GEVM, Tuareg Marketing Pvt. Ltd., Noida, Uttar Pradesh) and stored in airtight containers till further analysis. Figure 1 depicts the schematic depiction of the study design.

### 2.3 Nutritional analysis

The proximate composition of samples was analyzed using standard methods AOAC (11). *In vitro* protein digestibility was assessed by employing Akeson and Stahmann (12) method.

### 2.4 Anti-diabetic properties

The GI of grains was calculated *in vitro* by Goni et al. (4), and was expressed as the predicted glycemic index (pGI). The hydrolysis index (HI) was calculated by using the trapezoid rule to compute the area under the hydrolysis curves (AUC, 0–180 min). The percentage relationship between the AUC for the test food and the reference food (glucose) was used to calculate the HI. The technique described by Wongsa et al. (10) and Wang et al. (9) was employed to estimate the inhibitory activity of *α*-amylase and α-glucosidase.

### 2.5 Anti-nutritional properties

Trypsin inhibitor activity (TIA) is its ability to inhibit the caesinolytic activity of trypsin was determined by the method of Liu (13) and results were expressed as percent trypsin inhibition.

### 2.6 Statistical analysis

IBM SPSS Statistics 27.0 software, IBM, United States, was used for statistical analysis, and the data was reported as mean ± standard error (SE). Significant differences between means were found using analysis of variance (ANOVA) and *post-hoc* Duncan multiple range test (DMRT) at *p* < 0.05.

## 3 Results

### 3.1 Proximate composition

Nutritional analysis of samples BW, BM, and BS revealed significant differences (*p* < 0.05) in various parameters, shedding light on their respective nutritional qualities (Table 1). All samples stand out with notably good levels of ash, crude protein, crude fat, and physiological energy value (PEV). This suggested that these foods potentially offer a richer source of essential nutrients (both macro- and micro-nutrients) and energy. Additionally, BW and BM showcased higher carbohydrate content and lower crude fat in comparison to BS as shown in Table 1.

**Table 1:** Proximate composition of selected grains (black wheat, BW; barnyard millet, BM; black soybean, BS; % dw basis).

Sample	BW	BM	BS
Moisture (%)	7.76 ± 0.07^c^	9.43 ± 0.09^b^	11.87 ± 0.03^a^
Ash (%)	2.09 ± 0.01^c^	2.52 ± 0.05^b^	5.20 ± 0.06^a^
Crude Protein (%)	11.80 ± 0.15^b^	7.69 ± 0.11 ^c^	41.30 ± 0.28^a^
Crude Fat (%)	1.63 ± 0.03^c^	2.98 ± 0.00^b^	18.91 ± 0.09^a^
Total Carbohydrate (%)	76.72 ± 0.11^a^	77.37 ± 0.08^a^	22.72 ± 0.45^b^
PEV (kcal/100 g)	368.73 ± 0.25^b^	367.08 ± 0.45^c^	426.30 ± 0.16^a^

All values are Mean ± SE of three replicates on dry weight basis. ^a–c^values within the row with different superscript letters differed significantly (*p* < 0.05). Carbohydrate = 100 − (Moisture + Ash + Crude Protein + Crude fat). PEV, Physiological energy value.

### 3.2 Optimization of soaking and germination time

The relationship between soaking time and the weight gain of grains across three samples: BM, BS, and BW has been illustrated in Table 2. Initially, at 0 h of soaking, all samples start with a uniform weight of 50 g. As soaking time increased, there was a consistent trend of weight gain observed across all samples. However, BM exhibits the slowest rate of weight gain, while BS and BW showed comparatively higher rates, resulting in significant differences (*p* < 0.05) in weight gain between the samples. This disparity persisted throughout the soaking period, with BM consistently lagging behind BS and BW. Notably, beyond 8 h of soaking, the rate of weight gain tends to plateau for all samples, indicating that after 8, 12, and 16 h the weight of samples showed insignificant changes. Based on these observations, the optimum soaking time (OS) for BM, BS, and BW to achieve maximal weight gain was found to be around 12, 16, and 11 h where the weight was increased by 31, 130, and 57% respectively, beyond which was a insignificant change in weight.

**Table 2:** Optimization of soaking time for grains on the basis of weight gain.

Soaking time	Weight of soaked grain		
(h)	(g)		
	BM	BS	BW
0	50.00 ± 0.00^mA^	50.00 ± 0.00^nA^	50.00 ± 0.00^jA^
1	51.46 ± 0.06^lC^	60.14 ± 0.06^mA^	59.09 ± 0.01^iB^
2	52.76 ± 0.06^kB^	63.35 ± 0.06^lA^	63.22 ± 0.05^hA^
3	54.60 ± 0.03^jC^	65.78 ± 0.06^kB^	66.59 ± 0.01^gA^
4	55.29 ± 0.06^iC^	75.77 ± 0.06^jA^	69.29 ± 0.01^fB^
5	55.93 ± 0.03^hC^	77.95 ± 0.06^iA^	71.01 ± 0.03^eB^
6	57.37 ± 0.06^gC^	85.60 ± 0.07^hA^	72.71 ± 0.01^dB^
7	58.37 ± 0.06^fC^	92.86 ± 0.05^gA^	73.94 ± 0.01^cB^
8	62.34 ± 0.06^eC^	110.21 ± 0.06^fA^	75.71 ± 0.02^bB^
9	62.48 ± 0.06^dC^	110.80 ± 0.06^eA^	75.72 ± 0.01^bB^
10	62.49 ± 0.06^cC^	110.84 ± 0.06^dA^	75.72 ± 0.01^bB^
11	64.26 ± 0.06^bC^	111.58 ± 0.06^cA^	78.61 ± 0.06^aB^
12	65.31 ± 0.06^aC^	114.58 ± 0.05^bA^	78.62 ± 0.06^aB^
13	65.31 ± 0.06^aC^	114.58 ± 0.05^bA^	78.62 ± 0.06^aB^
14	65.31 ± 0.06^aC^	114.58 ± 0.05^bA^	78.62 ± 0.06^aB^
15	65.31 ± 0.04^aC^	114.58 ± 0.05^bA^	78.62 ± 0.06^aB^
16	65.31 ± 0.04^aC^	114.98 ± 0.05^aA^	78.63 ± 0.06^aB^
17	65.31 ± 0.05^aC^	114.98 ± 0.06^aA^	78.63 ± 0.06^aB^
18	65.31 ± 0.04^aC^	114.98 ± 0.06^aA^	78.63 ± 0.06^aB^
19	65.31 ± 0.04^aC^	114.98 ± 0.06^aA^	78.63 ± 0.06^aB^
20	65.31 ± 0.04^aC^	114.98 ± 0.06^aA^	78.63 ± 0.06^aB^

All values are Mean ± SE of three replicates on dry weight basis. ^A–C^values within the row with different superscript letters differed significantly (*p* < 0.05) where, A > B > C. ^a–n^values within the column with different superscript letters differed significantly (*p* < 0.05).

**Figure 2:**
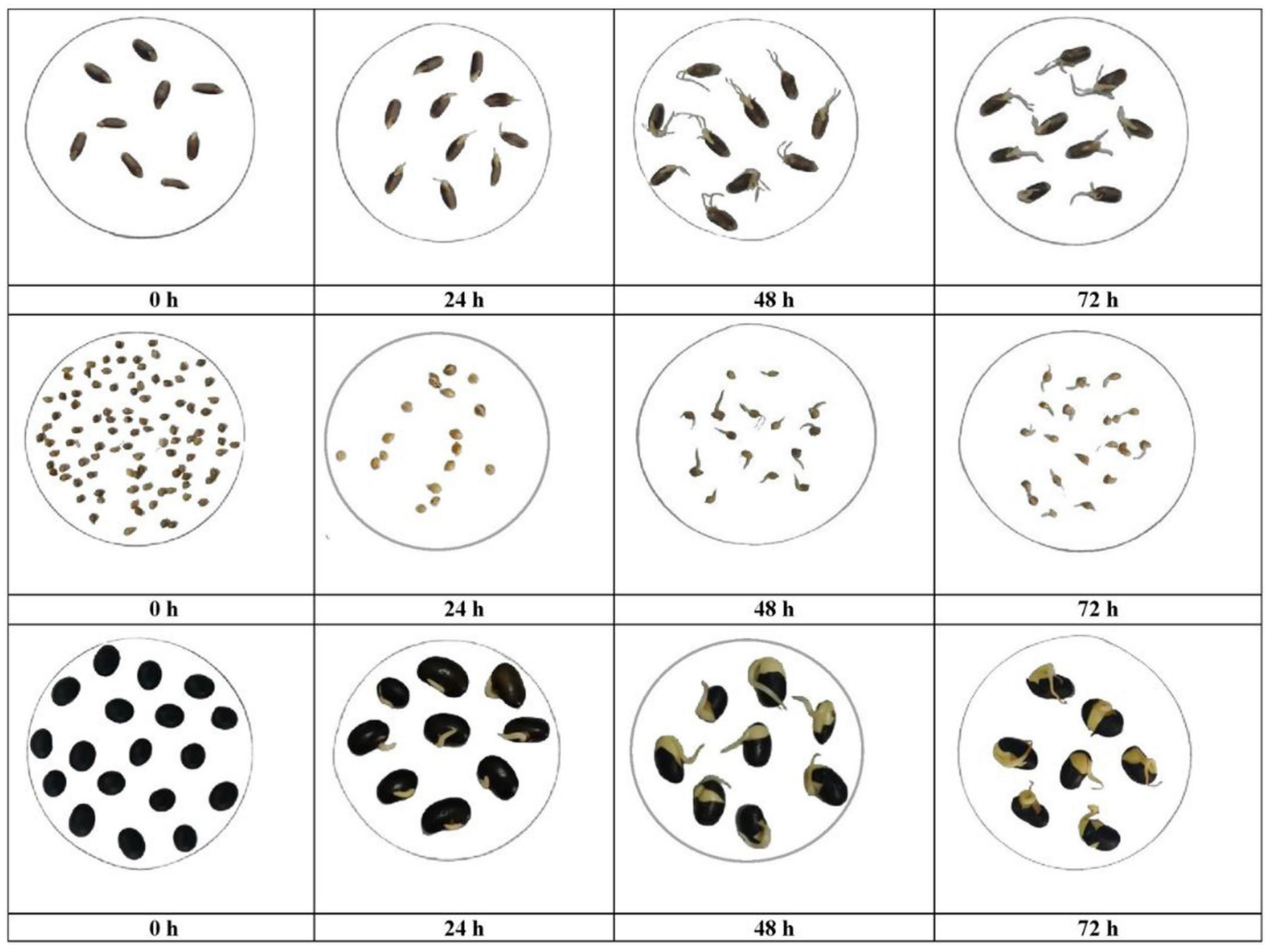
Effect of germination time on the sprout length of BW, BM and BS.

Germination was carried out for 72 h and was optimized based on sprout length (Figure 2). The data illustrates the relationship between germination time and sprout length across three samples: BW, BM, and BS. As the germination time increases, there are noticeable changes in sprout length for each sample. Initially, at 0 h, all samples show negligible sprout growth. However, after 12 h, BS exhibited significant (*p* < 0.05) sprout growth (6.00 ± 0.58 mm), while BM and BW remained relatively unchanged. By 24 h, all samples displayed varying degrees of sprout elongation, with BS exhibiting the longest sprouts (17.25 ± 0.14 mm), followed by BW (2.50 ± 0.00 mm) and BM (0.77 ± 0.02 mm). This trend continued up to 48 h, with BS consistently producing the longest sprouts (23.17 ± 0.44 mm), followed by BM (5.83 ± 0.17 mm) and BW (12.83 ± 0.17 mm). Notably, the rate of sprout growth stabilized after 48 h, as indicated by marginal changes in sprout length from 48 to 72 h across all samples. Adverse quality changes were noticed in the sprouts after 48 h of germination among all samples. These observations suggested that while germination time positively correlates with sprout length, there is a plateauing effect beyond a certain duration. Considering the above findings, the optimum germination time for all three samples was observed to be 48 h. However, these findings underscore the dynamic relationship between germination time and sprout growth, highlighting the potential for optimizing germination conditions to enhance sprout length, which can have implications for the improved nutritional quality and commercial viability of sprouted products.

### 3.3 Effect of processing on *in vitro* protein digestibility

**Figure 3:**
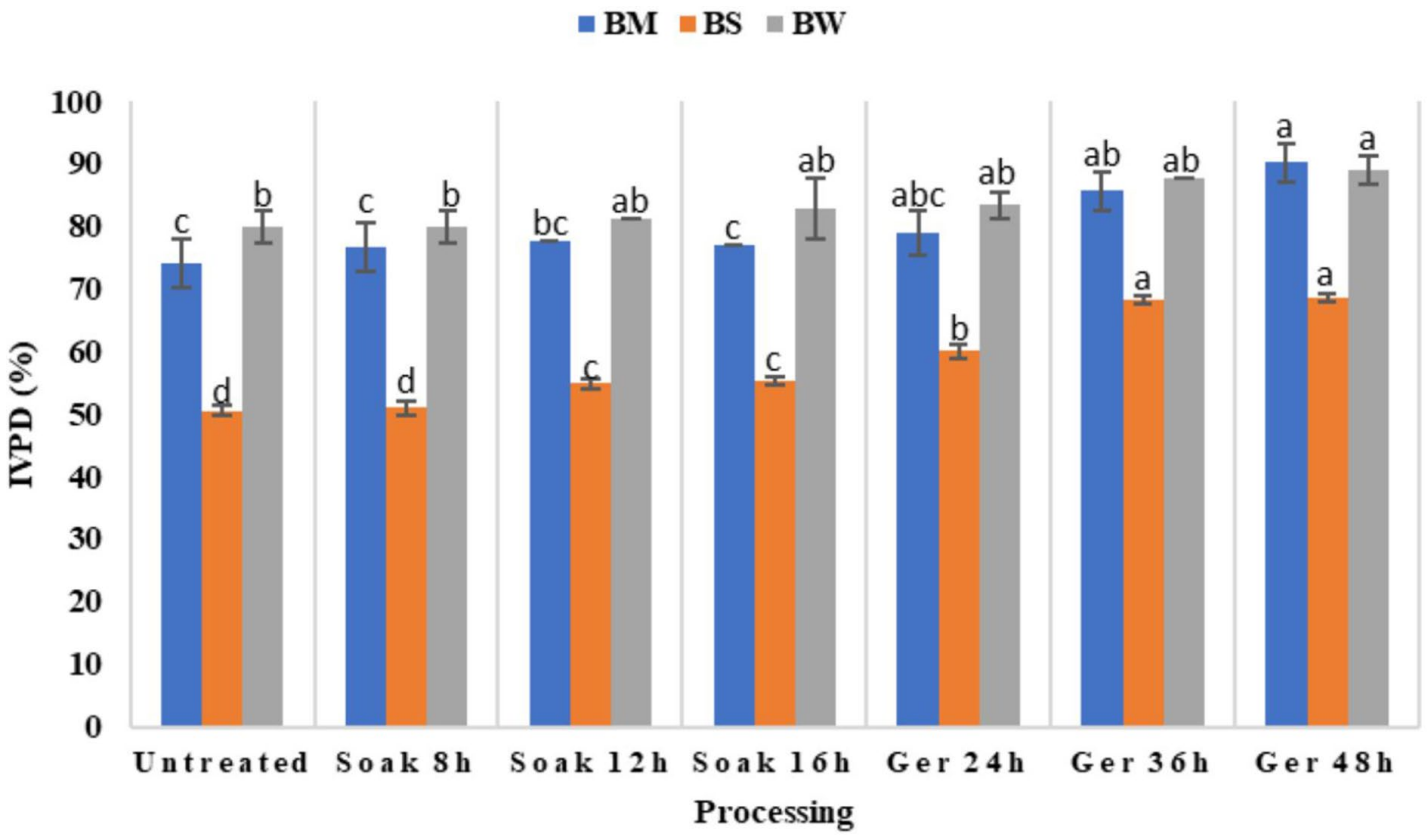
Effect of processing on protein digestibility of BW, BM and BS.

Impact of various processing methods on *in vitro* protein digestibility (IVPD) across three different samples viz., BW, BM, and BS has been illustrated in Figure 3. The results of the Duncan Multiple Range Test (DMRT) revealed that processing showed significant variations in IVPD at *p* < 0.05 (Table 3). Initially, untreated samples exhibited relatively lower IVPD values, with BS recording the lowest digestibility compared to BM and BW. However, after processing there was a notable improvement in IVPD across all samples. Soaking for 8 to 16 h lead to incremental increase in digestibility, with BM showing the highest enhancement after 12 h of soaking. Subsequent germination periods of 24 to 48 h resulted in significant (*p* < 0.05) enhancement in IVPD for all samples, with BM demonstrating the highest overall digestibility values. In the present study, protein digestibility improved significantly post-germination. Digestibility increased by 11.4% in NABIMG-11 (BW), 21.7% in VL 172 (BM), and 35.87% in VL Bhat 201 (BS; *p* < 0.05).

**Table 3:** Effect of processing on predicted glycemic index (pGI), *in vitro* protein digestibility (IVPD) and trypsin inhibitory units (TIU) of selected grains (black wheat, BW; barnyard millet, BM; black soybean, BS; % dw basis).

Raw material	BW						
Processing	Untreated	Soaked 8 h	Soaked 12 h	Soaked 16 h	Ger 24 h	Ger 36 h	Ger 48 h
p GI	44.34 ± 0.01^g^	44.50 ± 0.01^f^	47.24 ± 0.01^e^	48.02 ± 0.00^c^	47.34 ± 0.04^d^	49.34 ± 0.00^b^	49.55 ± 0.00^a^
IVPD (%)	79.90 ± 0.50^b^	79.90 ± 0.29^b^	81.38 ± 0.00^ab^	82.82 ± 0.36^ab^	83.42 ± 0.32^ab^	87.70 ± 0.00^ab^	89.05 ± 0.29^a^
TIU/mg	0.67 ± 0.01^a^	0.64 ± 0.00^b^	0.62 ± 0.00^c^	0.59 ± 0.00^d^	0.29 ± 0.00^e^	0.20 ± 0.00^f^	0.16 ± 0.00^g^
p GI	42.33 ± 0.00^g^	42.55 ± 0.02^e^	42.45 ± 0.01^f^	50.02 ± 0.01^b^	44.53 ± 0.00^d^	48.91 ± 0.01^c^	51.15 ± 0.00^a^
IVPD (%)	74.07 ± 3.90^c^	76.77 ± 3.84^c^	77.63 ± 0.00^bc^	77.06 ± 0.00^c^	78.99 ± 3.60^abc^	85.66 ± 2.96^ab^	90.18 ± 3.22^a^
TIU/mg	0.31 ± 0.00^a^	0.29 ± 0.00^b^	0.28 ± 0.00^c^	0.25 ± 0.00^d^	0.14 ± 0.00^e^	0.13 ± 0.00^f^	0.07 ± 0.00^g^
p GI	41.10 ± 0.01^g^	41.23 ± 0.00^f^	43.10 ± 0.01^e^	44.69 ± 0.00^d^	47.81 ± 0.04^c^	50.09 ± 0.01^b^	51.03 ± 0.06^a^
IVPD (%)	50.57 ± 0.71^d^	51.00 ± 1.18^d^	54.85 ± 0.67^c^	55.33 ± 0.67^c^	60.04 ± 1.20^b^	68.22 ± 0.66^a^	68.71 ± 0.66^a^
TIU/mg	76.23 ± 0.02^a^	71.13 ± 0.04^b^	68.42 ± 0.26^c^	67.18 ± 0.29^c^	57.47 ± 0.25^d^	43.83 ± 0.18^e^	23.17 ± 0.38^f^

All values are Mean ± SE of three replicates on dry weight basis. ^a–g^values within the row with different superscript letters differed significantly (*p* < 0.05) where, a > b > c.

### 3.4 Effect of processing on GI

**Figure 4:**
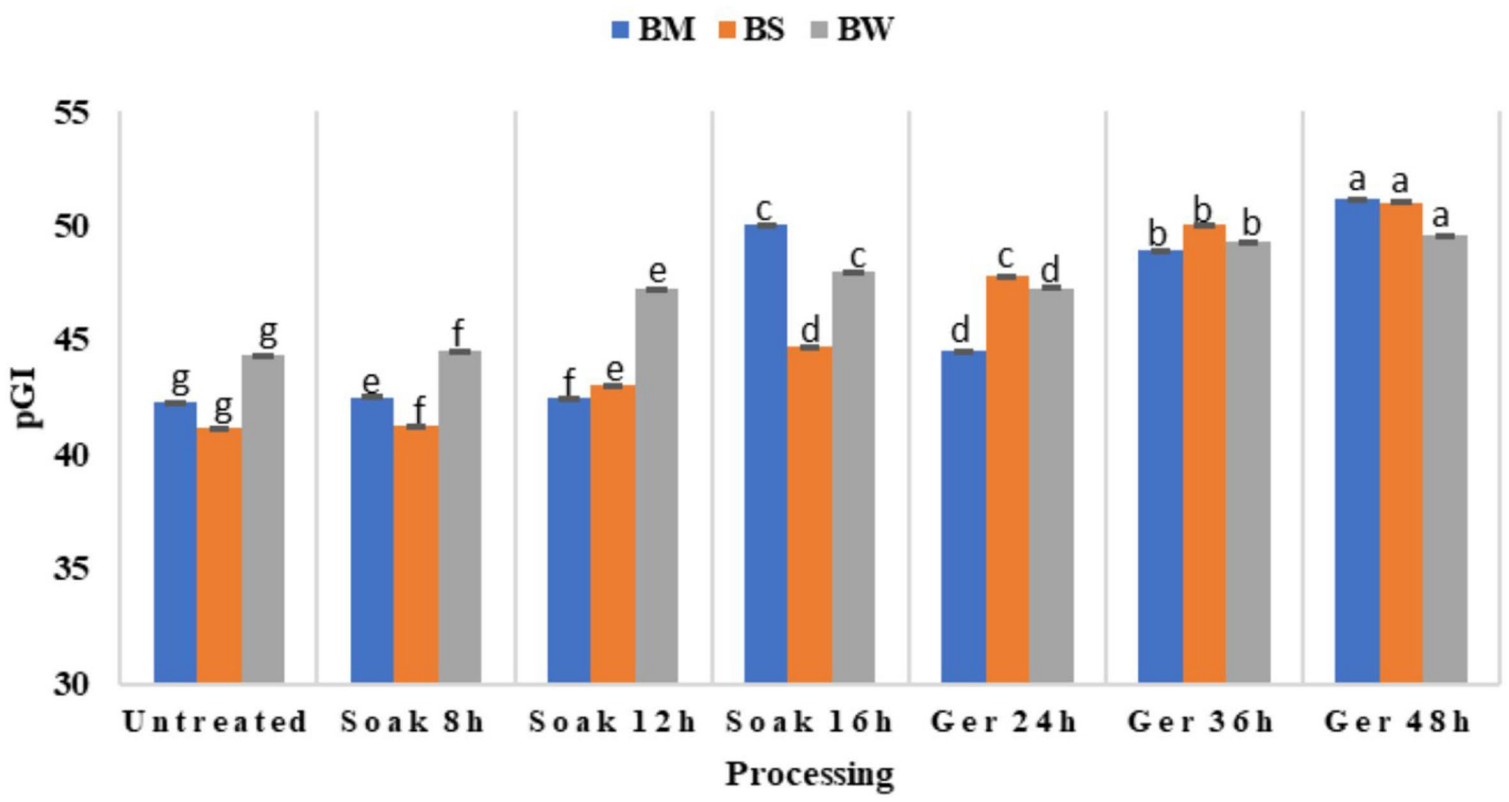
Effect of processing on the glycemic index of BW, BM and BS.

The pGI of grains differed significantly (*p* < 0.05) as a result of the processing of all three samples: BW, BM, and BS (Figure 4). Initially, untreated samples exhibited relatively low pGI values across all samples, with slight variation among them. Upon processing, there was a discernible trend of increasing pGI observed across all samples (Table 3). Soaking for 8 to 16 h led to marginal changes in pGI values, with fluctuations observed in all samples. However, more significant alterations were evident following germination periods of 24 to 48 h, with pGI values showing a substantial increase. Notably, BM displayed the most pronounced increase in pGI after 48 h of germination. BS showed a significant increase in pGI with the highest value of 51.03 after 48 h germination. When soaking prolonged to 16 h, BW and BM showed a significant increase in pGI (*p* < 0.05) however, when grains germinated for 10 and 11 h respectively, pGI showed a significant decline at 24 h germination, which was then gradually increased with increased in germination time.

### 3.5 Effect of processing on enzyme inhibitory activity

**Figure 5:**
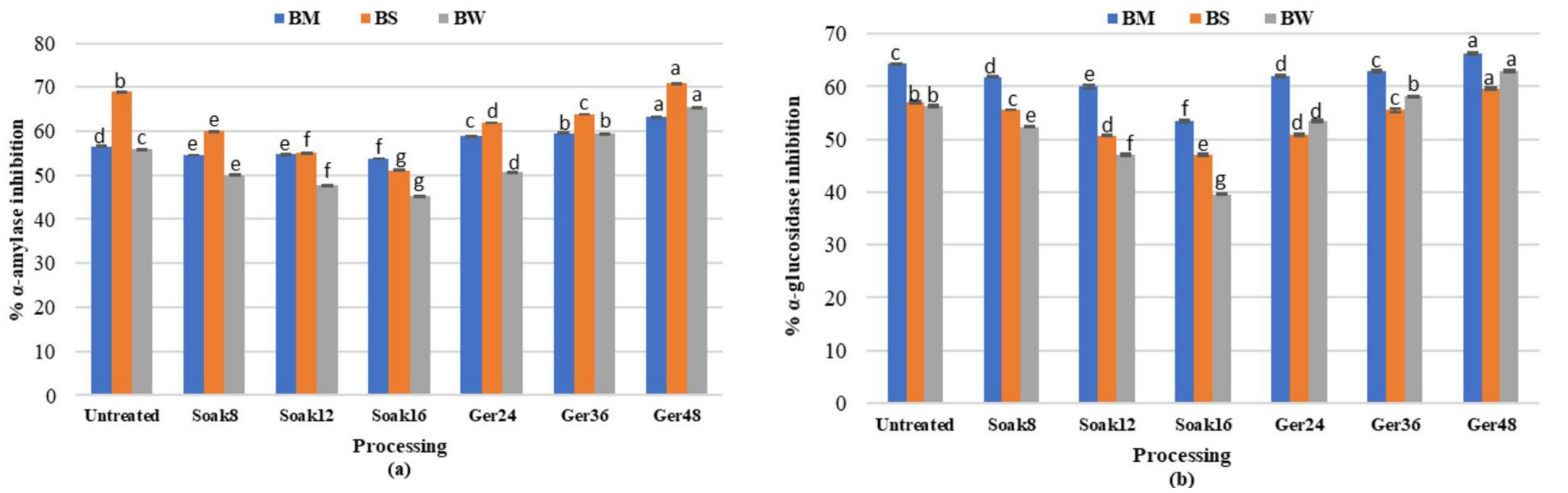
Effect of processing on **(a)** a-amylase and **(b)** a-glucosidase inhibitory activity of BW, BM and BS.

Alpha-amylase (1:2000 U/mg) inhibitory activity differed significantly (*p* < 0.05) in processed samples (BW, BM, and BS; Figure 5a). Soaking of grains reduced the *α*-amylase inhibition by 5, 18, and 26% for BM, BW, and BS, respectively. During germination, α-amylase inhibitory activity increased significantly (*p* < 0.05) with the increase in germination time. α-glucosidase (100 U/mg) inhibitory activity showed similar trends where soaking significantly reduced the α-glucosidase inhibition by 16, 29, and 17% for BM, BW, and BS, respectively, (Figure 5b). Whereas, germination significantly (*p* < 0.05) increased the inhibitory activity (α-amylase and α-glucosidase) with the increase in germination time by 17.06 and 11.8% for BW;11.8and 3.16% for BM (*p* < 0.01) respectively.

### 3.6 Effect of processing on trypsin inhibitory activity

**Figure 6:**
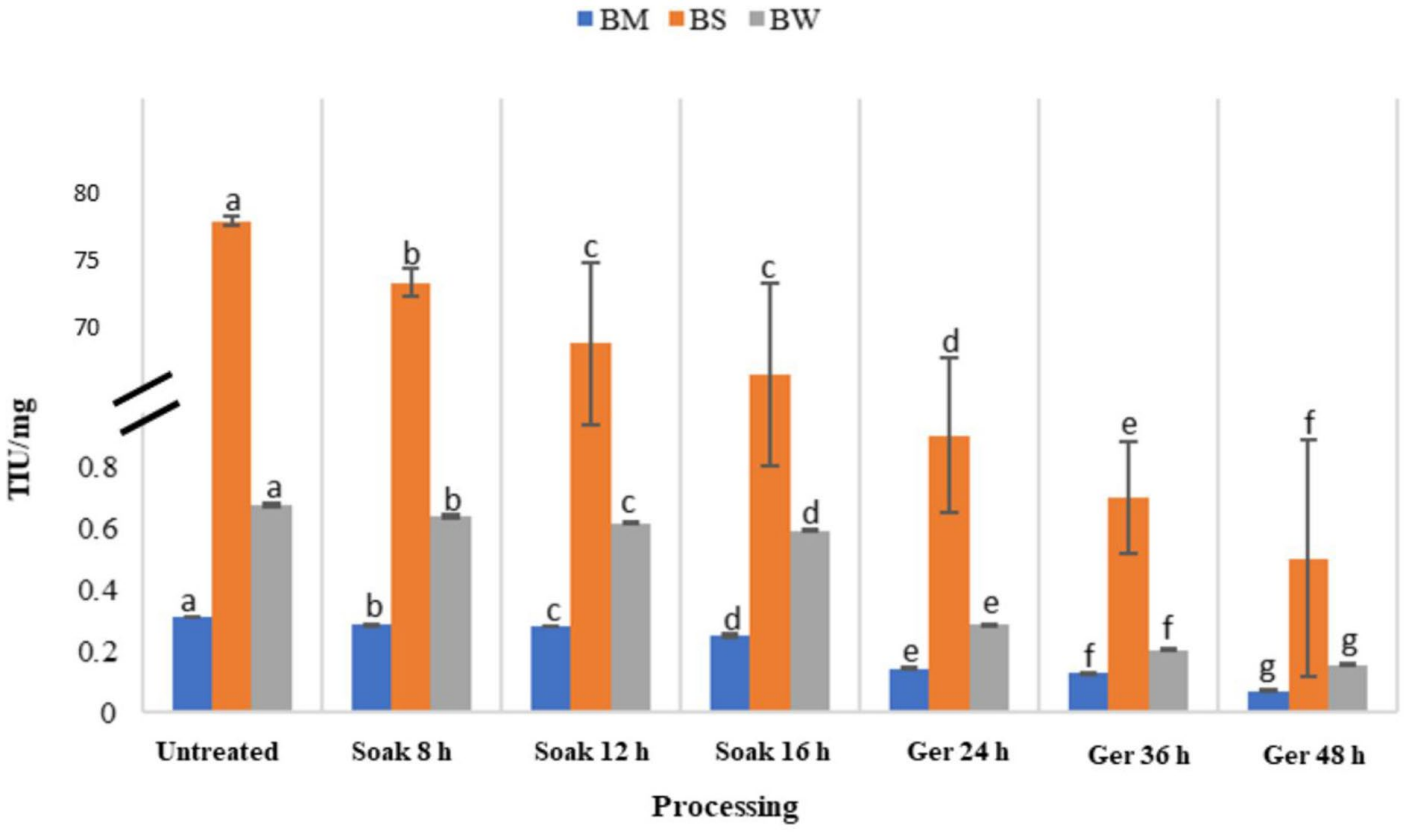
Effect of processing on trypsin inhibitory units in BW, BM and BS.

The impact of various processing methods on trypsin inhibitory units (TIU) in three different samples: BW, BM, and BS has been presented in Figure 6. Initially, untreated samples exhibited relatively high TIU values across all samples, with BS recording the highest inhibitory activity compared to BM and BW. Processing impacted a decrease in TIU values across all samples (Table 3). Soaking for 8 to 16 h led to gradual reductions in TIU, with a notable decrease of 19, 12, and 12% in BM, BW, and BS, respectively. Subsequent germination periods of 24 to 48 h resulted in a significant (*p* < 0.05) decrease in TIU by 77, 70, and 77% for BM, BW, and BS, respectively, with BM showing the most pronounced reduction in trypsin inhibitory activity.

### 3.7 Selection of processing method considering management of hyperglycemia

Opting for a suitable processing method is crucial for maximizing nutritional quality while minimizing glycemic response and trypsin inhibitory activity of the three low GI samples: BW, BM, and BS. BIS (14) standards, stated that the TIU/mg should be less than 75 for legume flour (soybean) that will be used in the development of products that require further cooking. Talking of the quality of protein in terms of its digestibility, Nosworthy et al. (15) reported that pulses with more than 70% protein digestibility are considered good in digestibility. As for the GI, it should be less than 55 to fall under the low-GI category (5). Therefore, considering these goals the processing method selected for the raw material was, BM (unprocessed), BS (germinated 48 h), and BW (unprocessed) as shown in Table 3. However, all three grains fall under the low GI category with GI < 55 before any bio-processing and even after the application of a bio-processing treatment that not only improved the protein digestibility but still showed lower GI.

## 4 Discussion

Dietary management plays a crucial role in maintaining blood glucose levels in diabetic individuals. This can be managed through slowing down rate of carbohydrate digestion by incorporating hydrolyzing enzymes (*α*-amylase and α-glucosidase) inhibitors or low GI foods in the diet or both. However, prior processing is essential to improve its nutritional and functional properties that affect the physical properties, nutritional composition, starch characteristics, cooking qualities, etc. Therefore, it is pivotal to understand those changes during processing to attain the maximum nutritional benefits from food.

### 4.1 Proximate composition

Proximate composition of raw grains emphasized the potential benefits of incorporating BW, BM, and BS individually or in combination into the diet for enhanced and balanced nutrient intake. All samples showed moisture content <12% which is indicative of their good shelf-life as increased moisture levels may contribute to reduced shelf-life due to greater susceptibility to microbial spoilage and oxidative degradation (16). Ash content in all samples was good, which denotes the mineral content of the food and consists of major elements such as Na, K, Ca, and Mg, and trace elements such as Fe, Zn, and Cu (7). Increased mineral concentration was linked, indirectly, to increased hygroscopicity through interactions between minerals carbohydrates, and water (17). Similar to other research, BW and BS showed a higher content of protein that represents the good quality of protein in terms of its amino acid composition (7, 18). Further exploration into the specific nutritional constituents of BW, BM, and BS could provide valuable insights into their potential dietary applications associated with various health conditions.

### 4.2 Optimization of soaking and germination time

Soaking increased weight gain due to water imbibition by grains leading to increased water absorption capacity during soaking which might be due to changes in total protein structure and loosening of starch polymers during soaking (19). Another explanation for weight gain after soaking may be because of the rise in the protein content which possesses both hydrophilic and hydrophobic nature and so may interact with both water and oil in foods, and ultimately accountable for enhancement in water absorption (20). However, further scientific discussion regarding the mechanisms underlying the saturation of grain absorption capacity and the potential implications for industrial processes and nutritional enhancement strategies is required. Germination time of grains positively correlated with sprout length. Many researchers studied the optimum germination time from 12 to 72 h (21, 22), some continued germination until the length of sprouts reaches 2/3rd of the length of seeds (23) associated with the specific grain type. However, in the present study, there observed a plateauing effect beyond a certain duration along with spoilage signs were observed in the samples on germination beyond 48 h. Studies reported that germination for a shorter duration is more effective for reducing antinutritional factors and preserving their chemical composition, thus improving their potential as a high-quality raw material for plant-based foods (24). These findings underscore the dynamic relationship between soaking-weight gain, and germination time-sprout growth, highlighting the need for optimizing soaking and germination conditions for BW, BM, and BS, which can have implications for the utilization of these grains in the development of value-added food products.

### 4.3 Effect of processing on *in vitro* protein digestibility

Processing like soaking and germination improved the digestibility of protein for all grains (*p* < 0.05). Similar observations were observed for cowpea and lentils (25); finger millet (26), and maize (22). These findings emphasized the efficacy of soaking and germination processes in enhancing protein digestibility, potentially attributed to enzymatic activities and structural modifications during processing (27).

Enhanced protein digestibility suggested enhanced biological value of proteins, which could be attributed to lower levels of antinutrients (28), the partial solubilization that occurs during seed sprouting, indicated by higher levels of water-soluble proteins and free amino acids in the sprouted seeds, and the proteolysis of complex protein structures into more digestible forms (22, 29). The improved digestibility, as observed in this study, may contribute to better protein and carbohydrate absorption, leading to enhanced satiety and reduced gastrointestinal discomfort. Enhanced protein digestibility can support muscle maintenance and metabolic health, while improved starch breakdown can minimize bloating and digestive issues, particularly for individual’s sensitivities to complex carbohydrates (29). However, the variations in digestibility among samples emphasize the importance of considering intrinsic factors such as grain type and composition in optimizing processing methods for improved protein bioavailability. Further exploration into the underlying mechanisms driving protein digestibility enhancements during processing could provide valuable insights for developing tailored strategies to maximize the nutritional benefits of grain-based products.

### 4.4 Effect of processing on GI

Findings suggested that processing methods, particularly prolonged germination durations, can significantly impact the glycemic response of grains (*p* < 0.05). The observed rise in pGI may be attributed to enzymatic activities during processing, which can potentially break down complex carbohydrates into simpler sugars (30, 31), leading to faster glucose release upon digestion. According to some studies, these circumstances also lead to both the leaching of bioactive components and increased enzyme activity (32). Additionally, variations in pGI among samples highlight the importance of considering grain type and processing conditions in managing glycemic responses, particularly for individuals with conditions like diabetes. In addition to dietary considerations, variations in the raw material’s structural qualities, the way food is processed, and the end product’s structure can all impact GI and insulin secretion (3). High GI of food resulted in rapid absorption of carbohydrates that evoked high postprandial blood glucose levels and insulin concentrations which lead to the development of insulin resistance and the incidence of T2D (31). Though, it is evident that even after prior processing, all the grains still fell under the low GI category (pGI < 55), further investigation into the underlying mechanisms driving changes in pGI during processing could yield valuable insights for optimizing grain processing techniques to mitigate adverse glycemic effects while maximizing nutritional benefits and establish their potential as a functional food.

### 4.5 Effect of processing on enzyme inhibitory activity

Enzyme inhibitory activity was affected significantly during processing. Soaking resulted in declined inhibition which aligns with previous research demonstrating that soaking reduces *α*-amylase and α-glucosidase activities (*p* < 0.05) (33). This decline in activity might be attributed to the leaching out of anthocyanin components that are responsible for imparting the *α*-amylase inhibitory activity (33, 34). However, the current study revealed that the grains, especially the germinated form, exhibited inhibitory activity against porcine pancreatic α-amylase and α-glucosidase because they had higher polyphenolic indexes (35), which are linked to the inhibition of hydrolyzing enzymes in a dose-dependent manner (36). According to recent research, phenolic compounds may bind to amino acid residues at the active sites of digestive enzymes through hydrogen bonding, which prevents the digestive enzymes from catalyzing the catalytic reaction on carbohydrates. This explains how phenolic compounds inhibit the activities of digestive enzymes (37, 38). Furthermore, these phenolic acids can bind to the active protein pocket of α-glucosidase, which decreases glucose absorption by favoring dissipation of the Na + electrochemical gradient. This is the mechanism that propels the build-up of active glucose and subsequent glucose transport (39), as a result of which they are recognized to reduce the absorption of glucose and control postprandial hyperglycemia (36), suggesting that they may be used therapeutically to treat diabetes mellitus. Though the *in vitro* demonstration of the inhibitory impact of the grain extracts has been demonstrated, these results suggest that the extracts may be able to reduce the starch hydrolysis-induced rise in postprandial glucose.

### 4.6 Effect of processing on trypsin inhibitory activity

Processing reduced the anti-nutritional factors, particularly prolonged germination durations, effectively reduced trypsin inhibitory activity in grains (*p* < 0.05) and thereby reduced the levels of anti-nutrients present. This reduction in TIU was comparable to the reduction observed in soybean (40), mung bean (41) and finger millet (22). The two factors responsible for this decrease in TIA are soaking in water, which causes soluble substances, such as trypsin inhibitors, to leach into the soaking water, and activating endogenous protease, which is related to trypsin inhibitor and is sensitive to temperature, water content, and other environmental factors (42). Activation of proteases resulted in the breakdown of proteins, including trypsin inhibitors, into smaller peptides and amino acids. This increased enzymatic activity during germination, leading to the degradation of trypsin inhibitors (22). Thus, the mechanism of endogenous proteases linked to trypsin inhibitor levels can be studied further. Additionally, variations in TIU reduction among samples highlight the importance of considering grain type and processing conditions in managing trypsin inhibitory activity, particularly for enhancing the nutritional quality and digestibility of grain-based products for maximizing nutritional benefits.

### 4.7 Selection of processing method considering management of hyperglycemia

Considering the requirements for the management of diabetes through dietary approaches, it was crucial to optimize the processing parameters as well as understand the effect of processing on the nutritional quality so that the end-product should impart maximum digestibility of protein with a minimum release of glucose into the bloodstream, along with minimum anti-nutrients to hinder nutrient bioavailability. Based on the standard values where IVPD should be more than 70% (15), GI should be less than 55 (5) and TIU should be less than 75 (14), it was observed that BW and BM do not require any prior processing to be utilized for the development of food products. However, for BS soaking followed by germination for 48 h proved to be an effective processing, that resulted in an IVPD of 68.706% and a GI of 51.03, with a TIU/mg of 23.166. While the TIU/mg value is slightly higher than the specified threshold, it remains within acceptable limits, considering the significant improvement in IVPD and GI. This suggests a potential benefit for managing postprandial glucose responses, making these bio-processed grains suitable for diabetic and pre-diabetic populations. Also, the global dietary landscape is increasingly shifting toward low-GI wholegrain foods due to their benefits in managing diabetes, promoting weight control, and supporting metabolic health (3). The observed reductions in GI and enzyme activity inhibition suggest that BW, BM, and BS could be formulated into functional foods targeting diabetic and pre-diabetic populations. This aligns with the growing consumer preference for functional foods that regulate postprandial glucose levels having potential applications including low-GI goods, fortified protein products, and diabetic-friendly meal alternatives, addressing the rising global incidence of metabolic disorders. Additionally, such techniques align with clean-label food trends by naturally improving food quality without synthetic additives (35).

## 5 Conclusion

The nutritional profiles of BW, BM, and BS are distinct, highlighting the potential benefits of incorporating these grains individually or in combination into the diet for enhanced and balanced nutrient intake. Optimizing soaking and germination times improved protein digestibility, GI, and, trypsin inhibitory activity, particularly across all samples. Considering the management of hyperglycemia, the selected processing methods for the raw materials were BM (unprocessed), BS (germinated 48 h), and BW (unprocessed), align with established standards and offer balanced nutritional benefits, highlighting the importance of tailored techniques for optimizing grain nutritional benefits. Soaking reduced *α*-amylase and α-glucosidase inhibition, while germination increased inhibitory activity.

Further exploration into the underlying mechanisms driving these changes during processing could inform more efficient grain processing techniques and enhance the nutritional quality of grain-based products.

## Data Availability

The original contributions presented in the study are included in the article/supplementary material, further inquiries can be directed to the corresponding author.
